# First Records of *Coquillettidia* (*Coquillettidia*) *richiardii* and *Culex* (*Culex*) *perexiguus/univittatus* (Diptera: Culicidae) Mosquitoes in Galicia (Northwest Spain)

**DOI:** 10.1155/japr/8881802

**Published:** 2025-05-12

**Authors:** María Isabel Silva-Torres, Yasmina Martínez-Barciela, Alejandro Polina González, Jose Manuel Pereira Martínez, Ánxela Pousa Ortega, Josefina Garrido González, Rita Sánchez-Andrade, María Sol Arias-Vázquez

**Affiliations:** ^1^Departamento de Patoloxía Animal, Grupo de Control de Parásitos (COPAR, GI-2120), Universidade de Santiago de Compostela, Lugo, Spain; ^2^Departamento de Ecoloxía e Bioloxía Animal, Universidade de Vigo, Vigo, Spain; ^3^Departamento de Zooloxía, Xenética e Antropoloxía Física, Universidade de Santiago de Compostela, Santiago de Compostela, Spain; ^4^Xunta de Galicia, Consellería de Sanidade, Santiago de Compostela, Spain

**Keywords:** *Coquillettidia*, *Culex*, mosquito surveillance, Northwest Spain, ReGaViVec

## Abstract

Three females of *Coquillettidia richiardii* (Ficalbi, 1889) and one female of *Culex perexiguus* Theobald, 1903/*Culex univitattus* Theobald, 1901, were recorded for the first time in Galicia (Northwest Spain) during entomological surveillance carried out by the regional vector surveillance network (ReGaViVec) between 2018 and 2022. The specimens were collected from a livestock farm in a *Csa* (hot Mediterranean summer) climatic zone and a private yard in a *Csb* (warm Mediterranean summer) climatic zone, according to the Köppen classification. Both species are recognized vectors of pathogens with medical and veterinary implications, underscoring the importance of recording their presence. This study documents their distribution in the region, examines the factors contributing to their detection, and highlights the need for ongoing and systematic vector surveillance.

## 1. Introduction

Over the last two decades, the emergence and re-emergence of mosquito-borne diseases, along with the introduction of invasive mosquito species, have become a major public health concern in Europe [1]. This phenomenon is linked to anthropogenic factors such as globalization and climate change. While the former has facilitated the spread of mosquito species and pathogens through increased travel and trade, the latter has created favorable conditions for mosquito development and pathogen transmission [2]. The mere presence of a competent vector in a region does not necessarily indicate active pathogen transmission, but it is an indispensable condition [3]. Therefore, detecting the presence and distribution of native and invasive mosquito species is one of the primary objectives of entomological surveillance programmes [4, 5].

The first entomological surveillance campaigns in Northwest Spain were conducted between 2005 and 2012 by the Galician government (Xunta de Galicia) and were focused on the detection of Bluetongue [6] and West Nile virus (WNV) vectors [7]. In 2017, following the recommendations of the World Health Organization for vector control [8] and the European Centre for Disease Prevention and Control [4, 5], the Galician government (Xunta de Galicia) established the ReGaViVec vector surveillance network in collaboration with the University of Santiago de Compostela (USC) and the University of Vigo (UVigo) to monitor the arrival of invasive mosquitoes and enhance the understanding of dipteran vector population in Galicia. So far, the ReGaViVec program has covered almost 300 sampling points across the region, updating the mosquito species inventory and detecting for the first time the invasive mosquito *Aedes albopictus* in collaboration with the citizen science platform Mosquito Alert [9, 10] (Table 1).

Until now, entomological studies have documented the presence and distribution of 24 mosquito species in Galicia, mostly belonging to the genera *Aedes*, *Anopheles*, *Culex*, and *Culiseta* (Table 1). Regarding the genus *Coquillettidia*, only two species are present in the Palearctic region: *Coquillettidia* (*Coquillettidia*) *buxtoni* (Edwards, 1923) and *Cq.* (*Coq.*) *richiardii* (Ficalbi, 1889) [15], both exhibiting a scattered distribution in Spain [16]. The presence of the genus *Coquillettidia* in Northwest Spain was first documented by Martínez-Barciela *et al.* [14] with the identification of *Cq. buxtoni* larvae. However, thanks to the continued efforts of the ReGaViVec entomological surveillance network, another two mosquito species are recorded for the first time in Galicia: *Cq. richiardii* and *Cx. perexiguus/univittatus*.

## 2. Materials and Methods

**Figure 1:**
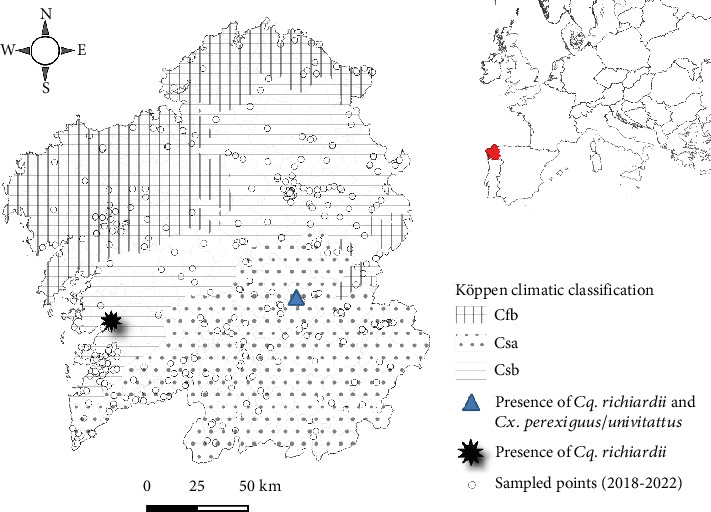
Köppen climatic classification for Galicia and geographical location of positive sampling points for *Cq. richiardii* and *Cx. perexiguus/univittatus*.

According to the Köppen climate classification [17], Galicia can be geographically divided into three climate zones: *Csa*—hot Mediterranean summer climate in the south, *Cfb*—oceanic climate in the north, and *Csb*—warm Mediterranean summer climate as a transitional zone between both [18]. The ReGaViVec vector surveillance network places different types of mosquito traps all over the territory (Figure 1). Selection criteria include potential host availability, trap accessibility, stagnant water and vegetation in the vicinity, favorable weather conditions for mosquito development, potential entry points of invasive species (e.g.: airports, ports, main roads…), and the outcomes of previous sampling efforts.

BG-Sentinel 2 traps equipped with BG-lure, BG-Pro traps baited with CO_2_ lure (Biogents AG Company, Regensburg, Germany), and miniature CDC light traps fitted with ultraviolet or white light (John W. Hock Company, Florida, United States) were used for adult mosquito trapping. Additionally, BG-GAT ovitraps (Biogents AG Company, Regensburg, Germany) and dipping techniques [19] were implemented for larvae collection. Specimens were identified to species level under a binocular magnifier and an optical microscope according to Becker et al. [15] European taxonomic keys. Entomological surveillance in this region was conducted from April or May to November. Adult traps were left running for 24 h, while ovitraps were kept in permanent positions. Traps were checked weekly or fortnightly depending on meteorological conditions and the surveillance relevance of each sampling point. Active sampling using the dipping technique was conducted sporadically during summer. Climatic variables on trap operational days were obtained from nearby weather stations at similar altitudes to the sampling site. Data was sourced from the Galician Meteorological website (https://www.meteogalicia.gal/), which provides free access to official weather station records from the Regional Autonomous Government.

## 3. Results

**Figure 2:**
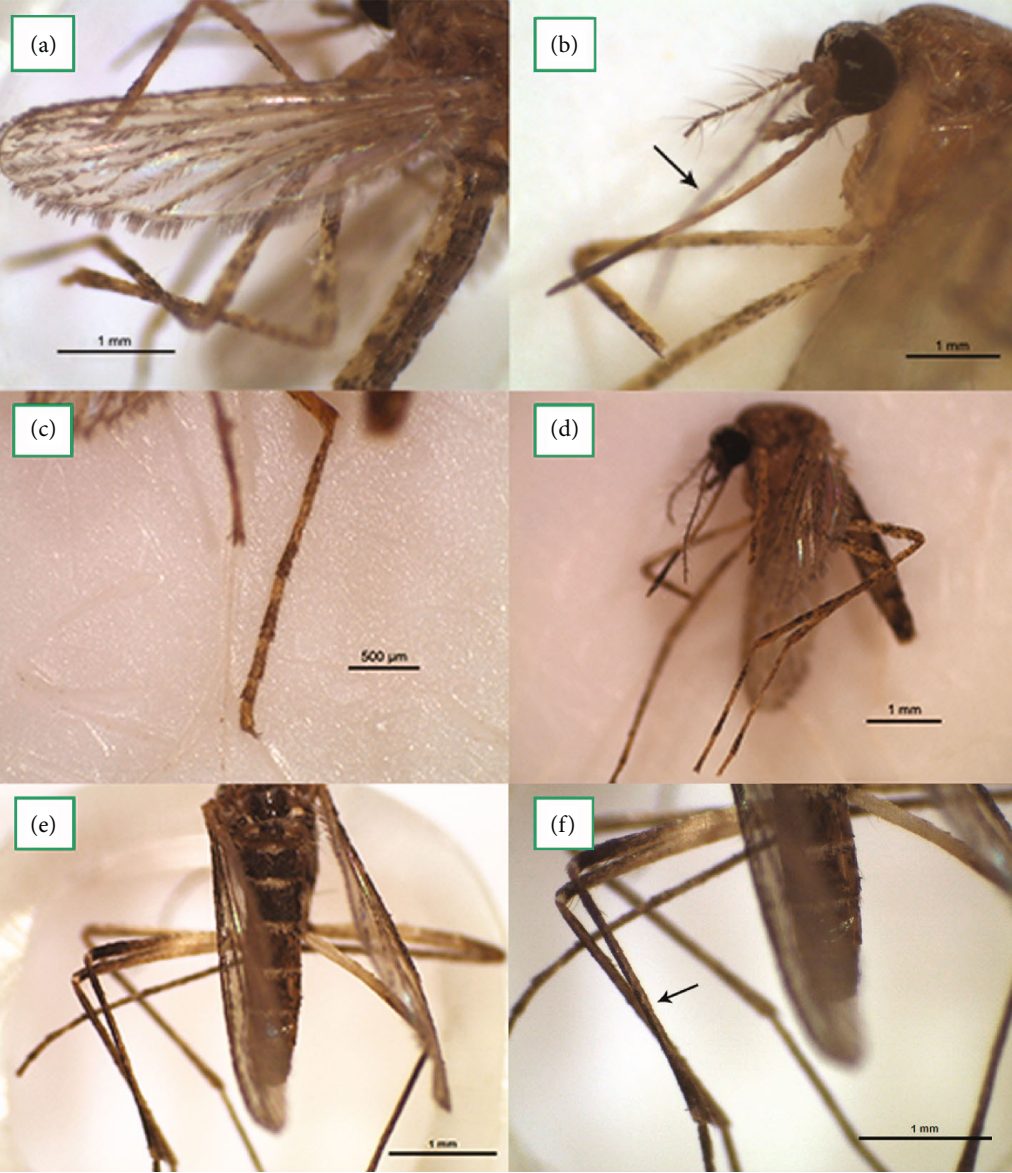
*Coquillettidia richiardii*: (a) Wing with arrow pointing to “leaf” shaped scales (2.5x). (b) Proboscis with dark apex and pale basal half (2x). (c) Tarsomere with pale rings (3x). (d) Yellowish and brown scale with mottled pattern on the femora and tibiae (1.6x). *Cx. perexiguus/univittatus*: (e) Abdominal terga with slightly convex pale basal pattern (2x). (f) Tibiae of hind legs with anterior pale longitudinal striae (2.5x).

During the entomological surveillance period of 2018–2022, three *Cq. richiardii* (Figures 2a, 2b, 2c, and 2d) and one *Cx. perexiguus/univittatus* (Figure 2e,f) females were identified.

The only specimen of *Cx. perexiguus/univittatus* was captured at a livestock farm in Monforte de Lemos at the end of July 2020, while *Cq. richiardii* was first detected at the same location by mid-August 2020 (Table 2). The farm is located within the *Csa* climate classification in the municipality of Monforte de Lemos, which belongs to the Ribeira Sacra winery region (42° 32⁣′ 32.107⁣^″^ N, 7° 31⁣′ 51.254⁣^″^ W), at an elevation of 333 masl (Figure 1). Livestock activities on the farm take place in a rural area 2.8 km from the town center, primarily focusing on ovine and bovine production, with less emphasis on porcine and poultry farming. Mosquitoes were captured using a CDC miniature light trap fitted with a 4 W UV light, placed indoors in a cattle enclosure near a stagnant body of marshy water.

The second and third *Cq. richiardii* females were collected in June and August 2022 in a private courtyard located in the coastal area of the municipality of Pontevedra (42° 26⁣′ 38.292⁣^″^ N, 8° 38⁣′ 36.448⁣^″^ W), at an elevation of 9 masl, characterized by a mild *Csb* climate (Figure 1). A BG-Pro trap baited with CO_2_ lure made from sugar, water, and yeast grains was placed outdoors in a periurban area close to the rivers Río Gándara and Río Lérez. Host availability included poultry and dogs.

The specimens from Monforte de Lemos farm were stored at the entomology collection of the COPAR group (Department of Animal Pathology, Faculty of Veterinary, USC), and those from the private courtyard in Pontevedra were kept in the BA2 Entomology Laboratory (Department of Ecology and Animal Biology, Faculty of Biology, UVigo).

## 4. Discussion

The detection of *Cq. richiardii* and *Cx. perexiguus/univittatus* is relevant for updating mosquito distribution records, as well as for assessing their potential vector role in Galicia.

Although *Cq. richiardii* is a common native mosquito in Europe, it exhibits a scattered distribution in Spain, being recorded across seven autonomous communities: Catalonia [20], Andalusia [16], and Community of Valencia [21], all with *Csa* climate; Castile and Leon [22], with *Csb* climate; and Aragon [23], La Rioja [24], and Basque Country [25], with *Cfb* climate. Most of these studies found this mosquito as a result of entomological surveillance conducted in wetlands. However, the presence of *Cq. richiardii* on a farm and in a courtyard near domestic animals aligns with previous research [26, 27]. This species has vector competence for WNV, Batai virus (BATV), Tahyna virus (TAHV), and the potential to transmit *Dirofilaria* spp. [28, 29]. Females exhibit flexible host selection patterns, making them potential bridge vectors for these pathogens [15]. Adult activity has been documented from late spring to late summer [29], and biting behavior occurs over a broad temperature range from 9°C to 26°C and relative humidity levels from 30% to 92% [15]. Additional years of observation are needed to establish a more precise seasonal activity period for this species in Galicia, but the favorable temperature and humidity conditions registered in the present study (Table 2) align with the weather conditions described for its optimal activity.

*Culex perexiguus* has been recorded predominantly in wetlands in southern *Csa* Spanish regions such as Andalusia [30, 31] and Extremadura [32], being documented only once in Northwest Spain [23]. Similar distribution has been observed for *Cx. univittatus* [16]. However, their exact geographic range and taxonomic classification in Spain have been constantly under review. The morphological differentiation of both species is challenging at all life stages, and its determination has often relied on distribution criteria [15]. *Culex univittatus* is primarily found in the Afrotropical region, particularly in the eastern and southern African subregion, whereas *Cx. perexiguus* is predominantly found in the Paleartic region, including southern Europe [15]. Encinas-Grandes [22] was the first to report *Cx. univittatus* in Spain, but it was not until a phylogenetic study by Mixão et al. [33] that its presence in the southern Iberian Peninsula could be confirmed. Since the specimen in the present study was identified using the taxonomic keys of Becker et al. [15], it will be referred to as *Cx. perexiguus/univittatus*.

In Southern Spain, *Cx. perexiguus* showed preference for natural areas over urban or suburban settings [34]. In the present study, *Cx. perexiguus/univittatus* was found in a cattle enclosure in a rural environment with suitable host availability. *Culex perexiguus* females exhibit flexible host-feeding behavior including birds and mammals; hence, this species can be considered a potential bridge vector for WNV, Usutu virus (USUV) and Sindbis virus (SINV) [5, 35]. The first two viruses have already been isolated from specimens captured in southern Spain [36].

The discovery of these two species in Galicia marks the second known occurrence of *Cq. richiardii* in the “Green Spain” northern region [25], which includes the Basque Country, Cantabria, Asturias, and Galicia; and the second north record of *Cx. perexiguus/univittatus* in Spain [16, 23], though this time in the western part of the country. *Coquillettidia richiardii* is not known for long-distance travel and typically remains close to its larval habitats [29]. Therefore, detecting this mosquito in two locations 90 km apart, combined with the lack of evidence of its presence in surrounding regions [16], suggests that it is a native species with a scattered and limited distribution in Galicia. On the other hand, *Cx. perexiguus*/*univittatus* presence in areas bordering Galicia has not been documented [16] and results from other studies are difficult to extrapolate due to their focus on wetland habitats. The sporadic capture of a single *Cx. perexiguus/univittatus* female during this period confirms its presence but complicates the assessment of its seasonal activity, population size, and distribution. Continued efforts in vector surveillance and control are therefore essential, especially since this mosquito is a recognized vector for pathogens of both veterinary and human health significance.

As Bakran-Lebl et al. [37] stated, the discovery of new species in a region raises questions about why they were not detected earlier, and their conclusions can be extrapolated to this study. One reason could be the limitations of previous monitoring and surveillance efforts, which were primarily focused on assessing the risk of Bluetongue virus [6] and WNV transmission in Galicia [7]. This targeted approach may have resulted in a narrower focus within the territory. In contrast, ReGaViVec covers most of the territory and is aimed at assessing the risk of vector-borne disease transmission in the region by studying the distribution, diversity, and ecology of vectors of medical and veterinary importance. Since its creation, the efforts of this ambitious regional surveillance program have expanded each year, increasing from 12 trapping sampling points in 2017 to 84 in 2022.

Another explanation could be the use of inadequate trapping methods, which may have led to an underestimation of the mosquito population or its range. The most effective traps for capturing *Cq. richiardii* adults are CO_2_-baited traps [5], but the ReGaViVec vector surveillance started using them in 2022, which matches the second and third female captures. The efficacy of CO_2_-baited or light traps in capturing *Cx. perexiguus* adults remains unclear [5]; nonetheless, in this study, the female was captured using a CDC UV light trap. No larvae of *Cq. richiardii* or *Cx. perexiguus/univittatus* were captured using the conventional dipping technique for mosquito sampling [19]. The absence of *Cq. richiardii* larvae could be attributed to the omission of the *scraping dipping technique* recommended for this species, which dislodges anchored larvae from submerged aquatic plants and enhances their detection [5]. *Coquillettidia richiardii* has a slow development [29], and it is more likely to be found at stagnant or permanent water bodies with vegetation cover [38]. Similarly, *Cx. perexiguus* and *Cx. univittatus* can be found in different stagnant water bodies, including clean to moderately polluted swamps with vegetation cover [15]. Following these criteria, sampling the swampy area near the Monforte de Lemos farm would have been justified to confirm the presence of immature stages of both species. However, inaccessibility prevented sampling at this site, restricting the study to adult mosquito collection.

The reliance on limited sampling scope and methods, along with ecological factors such as low population densities and specific habitat preferences, may have contributed to overlooking *Cq. richiardii* and *Cx. perexiguus/univittatus* populations in the past. Identifying these species for the first time underscores the success of expanding ReGaViVec surveillance efforts and highlights the importance of comprehensive and sustained surveillance. However, further research is needed to better define the distribution and phenology of these mosquitoes in Galicia. Although the potential risk of pathogen transmission by *Cq. richiardii* and *Cx. perexiguus/univittatus* in this region remains uncertain, the possible role of the former as a vector of *Dirofilaria immitis* emphasizes the need to include *Cq. richiardii* in future pathogen screenings, especially in the Pontevedra region, where this study confirmed the presence of this species and previous studies have shown a high prevalence of the parasite [39].

## 5. Conclusions

The detection of *Cq. richiardii* and *Cx. perexiguus/univittatus* expands the known mosquito inventory in the Galician region. These species are recognized vectors of pathogens that affect human and animal health. Therefore, the findings of this study underscore the critical need for continued monitoring and research to improve risk assessment of these mosquito species in Galicia. Moreover, strengthening surveillance efforts will be essential for the early detection of mosquito species and timely response to emerging vector-borne diseases in the region.

## Figures and Tables

**Table 1:** Updated list of the 26 mosquito species identified in Galicia.

***Anopheles***	***Aedes***	***Culex***	***Coquillettidia***	***Culiseta***
*An. claviger* ^b^	*Ae. albopictus* ^ah^	*Cx. hortensis* ^b^	*Cq. buxtoni* ^g^	*Cs. annulata* ^b^
*An. maculipennis s.l.* ^b^	*Ae. caspius* ^b^	*Cx. impudicus* ^g^	*Cq. richiardii* ^a*Δ*^	*Cs. fumipennis* ^g^
*An. petragnani* ^i^	*Ae. geniculatus* ^d^	*Cx. mimeticus* ^i^		*Cs. longiareolata* ^b^
*An. plumbeus* ^af^	*Ae. pullatus* ^c^	*Cx. modestus* ^c^		*Cs. morsitans* ^g^
	*Ae. vexans* ^b^	*Cx. perexiguus* ^a*Δ*^		*Cs. subochrea* ^b^
	*Ae. vittatus* ^e^	*Cx. pipiens* ^b^		
		*Cx. territans* ^g^		
		*Cx. theileri* ^b^		
		*Cx. torrentium* ^af^		

^a^Species detected for the first time under the ReGaViVec surveillance efforts.

^b^Lucientes et al. [6].

^c^Lucientes et al. [7].

^d^Vieira-Lanero et al. [11].

^e^Eritja et al. [12].

^f^Martínez-Barciela et al. [13].

^g^Martínez-Barciela et al. [14].

^h^Martínez-Barciela et al. [9].

^i^Martínez-Barciela et al. [10].

^*Δ*^Species detected during 2018–2022 as a result of the present study.

**Table 2:** Detailed information for captures including location, type of premise, trapping dates, number of collected specimens (*N*), Köppen classification, and climatic values recorded (MaxMean *T*^a^: maximum mean temperature; MinMean *T*^a^: minimum mean temperature; MeanRH: mean relative humidity).

**Species**	**Location**	**Premise**	**Dates**	**N**	**Köppen classification**	**Mean **T**^a^ (°C)**	**MaxMean **T**^a^ (°C)**	**MinMean **T**^a^ (°C)**	**MeanRH (%)**
*Cx. perexiguus/univittatus*	Monforte de Lemos	Livestock farm	29-30/VII/2020	1	Csa	21.2	29.6	18.5	85
*Cq. richiardii*	Monforte de Lemos	Livestock farm	11-12/VIII/2020	1	Csa	19.4	26.2	15.6	83.5
	Pontevedra	Private courtyard	02-03/VI/2022	1	Csb	18.1	23.1	13.4	74.9
	Pontevedra	Private courtyard	04-05/VIII/2022	1	Csb	24.1	31.3	19.1	57.1

## Data Availability

The data that support the findings of this study are available from the corresponding author upon reasonable request.
